# Primary signet-ring cell carcinoma of the urinary bladder ‒ A rare bladder tumor

**DOI:** 10.1016/j.clinsp.2024.100500

**Published:** 2024-09-23

**Authors:** Xin Hong, Tian Wang, Qing Liu, Jianlong Bi, Hui Li

**Affiliations:** a Department of Urology, Peking University International Hospital, Beijing, China; b Department of Emergency, Peking University International Hospital, Beijing, China

**Keywords:** Primary signet-ring cell carcinoma, Bladder adenocarcinoma, GATA3, Mortality, Independent risk factor

## Abstract

**Aim:**

Primary Signet Ring Cell Carcinoma (SRCC) of the bladder accounts for only 1%‒4% of all bladder malignancies. To date, few studies have been conducted to investigate the characteristics of SRCC. This study aimed to investigate the clinical features and treatments of SRCC and explore the independent risk factors of survival in SRCC patients.

**Patients and methods:**

A retrospective study was conducted on 32 eligible patients. The survival rate was calculated with the Kaplan-Meier method, and the COX proportional hazards model was used to investigate the independent risk factors of prognosis.

**Results:**

In the present study, the 1-year and 2-year survival rates of SRCC patients were 53.1% and 9.4%, respectively. The TNM stage, tumor differentiation, and metastasis after treatment were risk factors for the prognosis of SRCC patients (p < 0.05), while surgical treatment, chemotherapy, and positive GATA3 expression were protective for prognosis (p < 0.05). Multivariate analysis showed that GATA3 was an independent protective factor for prognosis (p < 0.05), and T-stage was an independent risk factor (p < 0.05).

**Conclusions:**

Primary SRCC of the bladder is highly malignant and has a poor prognosis. Its clinical and imaging findings are usually non-specific. Early radical cystectomy and postoperative adjuvant systemic chemotherapy are helpful to improve the survival rate. T-stage is an independent risk factor for survival, and positive GATA3 expression is protective for primary SRCC of the bladder.

## Highlights

• Primary SRCC of the bladder has no specific clinical manifestations at early stage.
• GATA3 serves as an independent protective factor for primary bladder SRCC.
• Radical cystectomy is the treatment of choice for primary bladder SRCC.

## Introduction

Adenocarcinomas of the urinary system are extremely rare, and bladder adenocarcinomas account for only about 0.5% to 2% of all bladder malignancies.^1^ In most patients, the Signet Ring Cell Carcinoma (SRCC) of the bladder as a bladder adenocarcinoma usually results from the metastasis of gastric cancer, colon cancer or breast cancer. Primary SRCC of the bladder was first reported by Saphir in 1955,^2^ and it accounts for only 1%‒4% of all bladder malignancies.^3^ SRCC of the bladder can be found in patients at any age, and its incidence gradually increases in subjects older than 40, with a male-to-female ratio of 2.70‒3.20:1.^3^ The most common manifestation of SRCC of the bladder is macroscopic hematuria, followed by symptoms of urinary tract irritation (frequent micturition, urgent micturition, odynuria, lower abdominal discomfort, etc.). Some patients may have muccitic urine, the amount of muccitic urine varies, and the thick mucus can also block the urethra, causing urinary retention, which is one of the characteristics of SRCC of the bladder.^4^

Primary SRCC of the bladder at an early stage shows submucosal infiltrative growth, and clinical symptoms are often present at a late stage. Moreover, cystitis and metaplasia usually precede the symptoms of the bladder and thus SRCC of the bladder is often diagnosed only when it infiltrates into the muscle layer.^5^ Primary SRCC of the bladder displays hypoechoic areas and localized bladder wall thickening on ultrasonography. Computerized Tomography (CT) can accurately determine the tumor size, bladder wall thickness, extent of bladder invasion and lymph node metastasis. SRCC of the bladder at an early stage usually manifests as diffuse bladder wall thickening, and thus cystoscopy often fails to identify the lesions in some patients. Therefore, a biopsy of the full-thickness bladder is usually required for the clinical diagnosis.^6^

The survival rate of SRCC patients is usually very low because it is often diagnosed at an advanced stage. It has been reported that the average 5-year survival rate is 27%‒30% for SRCC patients,^7^^,^^8^ about 1/4 of SRCC patients are found to have distant metastases at diagnosis, and about 60% of patients die within one year after diagnosis.^9^ Therefore, it is of great clinical significance to clarify the clinical characteristics, histological features, treatments, immunohistochemical markers, and independent risk factors for the prognosis of patients with SRCC of the bladder.

## Materials and methods

A total of 32 patients meeting the inclusion criteria and receiving treatments between January 2010 and October 2021 were included in this retrospective study. They were diagnosed with primary SRCC of the bladder. The age, gender, main complaints, cystoscopic findings (tumor location), grade, TNM stage, treatments, immunohistochemical findings, and follow-up findings were recorded for further analysis. This study was conducted in accordance with the STROBE Statement rules. This study was approved by the ethics committee of Peking University International Hospital (2024-026[BMR]).

Inclusion criteria were as follows: (1) Pathological examination after biopsy by cystoscopy or surgical resection confirmed the diagnosis of SRCC. (2) Tumors of the digestive system were not identified on contrast-enhanced CT of the abdomen and pelvis and on gastrointestinal endoscopy. (3) The tumor grade and stage were known. (4) The treatments and follow-up data were complete.

The exclusion criteria were as follows: (1) The space-occupying lesions in the bladder were not diagnosed as SRCC on pathological examination. (2) SRCC was also found in the digestive system in patients with SRCC of the bladder. (3) Although pathological examination showed SRCC of the bladder, data about treatments and follow-up were incomplete.

### Immunohistochemistry

Tissues were collected, embedded in paraffin, and sliced into sections (4 μm in thickness). The sections were heated at 70°C for 2h and then allowed to cool at room temperature for further use. These sections were successively treated with xylene for 3 min, absolute ethanol for 3 min, 95% ethanol for 3 min, 85% ethanol for 3 min and 75% ethanol for 3 min. After rinsing with flowing water for 3 min and Phosphate Buffered Saline (PBS) thrice (2 min for each), sections were subjected to antigen retrieval in citrate buffer for 10 min, then allowed to cool at room temperature for 1‒2h, and rinsed with PBS 3 times (2 min for each). Sections were treated with 3% hydrogen peroxide for 15 min at room temperature in the dark to inactivate endogenous peroxidase and then rinsed with PBS 3 times (2 min for each). After incubation overnight with primary antibody at 4°C, sections were incubated with secondary antibody for 1h, followed by DAB staining. For PAS staining, sections were treated with periodic acid for 10 min, Schiff solution for 15‒30 min in the dark, and hematoxylin for nuclear staining. After dehydration and transparentization, sections were mounted with neutral gum. Antibodies used in this study included GATA3, Ki-67, Carcinoembryonic Antigen (CEA), RKT7, RKT20, villin, AB-PAS, Vascular Endothelial Growth Factor (VEGF), p53, E-cadherin, CDX2, Estrogen Receptor (ER), and Progesterone Receptor (PR).

### Statistical analysis

Statistical analysis was performed with SPSS version 19.0. The Kaplan-Meier method was used for the analysis of the survival rate. The log-rank test was employed to analyze the factors affecting survival time, and the survival was compared among subgroups. The prognostic value was assessed with the COX proportional hazards model. A value of p < 0.05 was considered statistically significant. When the factors were related to the survival in the univariate analysis, they were included in multivariate analysis, aiming to identify the independent risk factors affecting the survival time of patients with SRCC of the bladder.

## Results

### General characteristics

Among 32 patients, there were 22 males and 10 females with a mean age of 61.03±7.39 years (range: 48‒73 years). Macroscopic haematuria was found in 19 patients, irritative symptoms in 8, and anuric symptoms in 5. Urine cytology showed positive results in 20 patients, negative results in 7, and unknown results in 5. The tumor was found in the trigone of the bladder in 5 patients, a dome of the bladder in 6, anterior wall of the bladder in 2, lateral wall of the bladder in 6, the bladder neck in 3, and posterior wall of bladder in 3, and the overlapping lesion was observed in 7 patients. In addition, moderate differentiation was observed in 2 patients, poor differentiation in 26 and undifferentiation in 4. According to the TNM staging, T2 stage was found in 3 patients, T3 stage in 15 and T4 stage in 14; N0 stage was found in 15 patients, N1 stage in 8, N2 stage in 7 and N3 stage in 2; M0 stage was found in 22 patients and M1 stage in 10. To date, no consensus has been developed for the treatment of SRCC of the bladder, and thus the treatments were different in these patients. The treatments included radical cystectomy alone, radical cystectomy plus chemotherapy, radical cystectomy plus radiotherapy, partial cystectomy alone, partial cystectomy plus chemotherapy, and Transurethral Resection of Bladder Tumor (TURBT) plus radiotherapy. In addition to surgical treatment, chemotherapy was a common treatment, and gemcitabine and platinum-based protocols were used in the chemotherapy of most patients. It has been reported that the combination of methotrexate, vincristine, doxorubicin, cisplatin, gemcitabine, and 5-Fluorouracil (5-FU) may have therapeutic effects on primary SRCC of the bladder to a certain extent.^10^ In clinical practice, surgery in combination with one or more treatments (including chemotherapy and radiotherapy) is a widely accepted strategy that may achieve a better efficacy and prolong the disease-free survival time. During the follow-up period, 10 patients had no metastasis, and metastasis was diagnosed in 22 patients. The lung, liver, bone, meninges, and omentum were the most common sites of metastasis. In addition, the survival time of 17 patients was longer than 1 year, 2 were still alive with the disease, 27 died of this disease, and 3 were still alive without disease (Table 1).

**Table 1:** General characteristics and treatments of patients with primary SRCC of the bladder.

**Characteristic**		**Cases** **(n = 32)**	**Percentage (%)**
Age(year)	> 60		
	≤ 60		
Gender	Male	22	69.00%
	Female	10	31.00%
Main complaints	Macroscopic hematuria	19 (59)	59.00%
	Irritative symptom	8	25.00%
	Anuric symptom	5	16.00%
Urine cytology	Positive	20	63.00%
	Negative	7	22.00%
	Unkown	5	15.00%
Tumor location (%)	Trigone	5	16.00%
	Dome	6	19.00%
	Anterior wall	2	6.00%
	Lateral wall	6	19.00%
	Bladder neck	3	9.00%
	Posterior wall	7	22.00%
	Overlapping lesion	3	9.00%
Grade (%)	Moderately	2	6.00%
	Poorly	26	81.00%
	Undifferentiated	4	13.00%
T stage	T1	0	0.00%
	T2	3	9.38%
	T3	15	46.88%
	T4	14	43.75%
N stage	N0	15	46.88%
	N1	8	25.00%
	N2	7	21.88%
	N3	2	6.25%
M stage	M0	22	68.75%
	M1	10	31.25%
Treatment	Radical cystectomy	4	12.50%
	Radical cystectomy+Chemotherapy	5	15.63%
	Radical cystectomy+Radiation	4	12.50%
	Partial cystectomy	2	6.25%
	Partial cystectomy+Chemotherapy	4	12.50%
	TURBT+Radiation	2	6.25%
	Chemotherapy	11	34.38%
Metastases	None	10	31.25%
	Lung	7	21.88%
	Liver	6	18.75%
	Spine	3	9.38%
	Meninges	1	3.13%
	Omentum	1	3.13%
	≥2 organs	4	12.50%
Immunohistochemistry	GATA-3 positive (%)	23	71.88%
	Ki-67 positive (%)	20	62.50%
	CEA positive (%)	14	43.75%
	AB-PAS positive (%)	11	34.38%
	RKT20 positive (%)	9	28.13%
	Villin positive (%)	8	25.00%
	VEGF positive (%)	5	15.63%
	p53 positive (%)	6	18.75%
Follow-up period	AWOD	3	9.38%
	DOD	27	84.38%
	AWD	2	6.25%

### Histological and immunohistochemical examinations

**Fig. 1:**
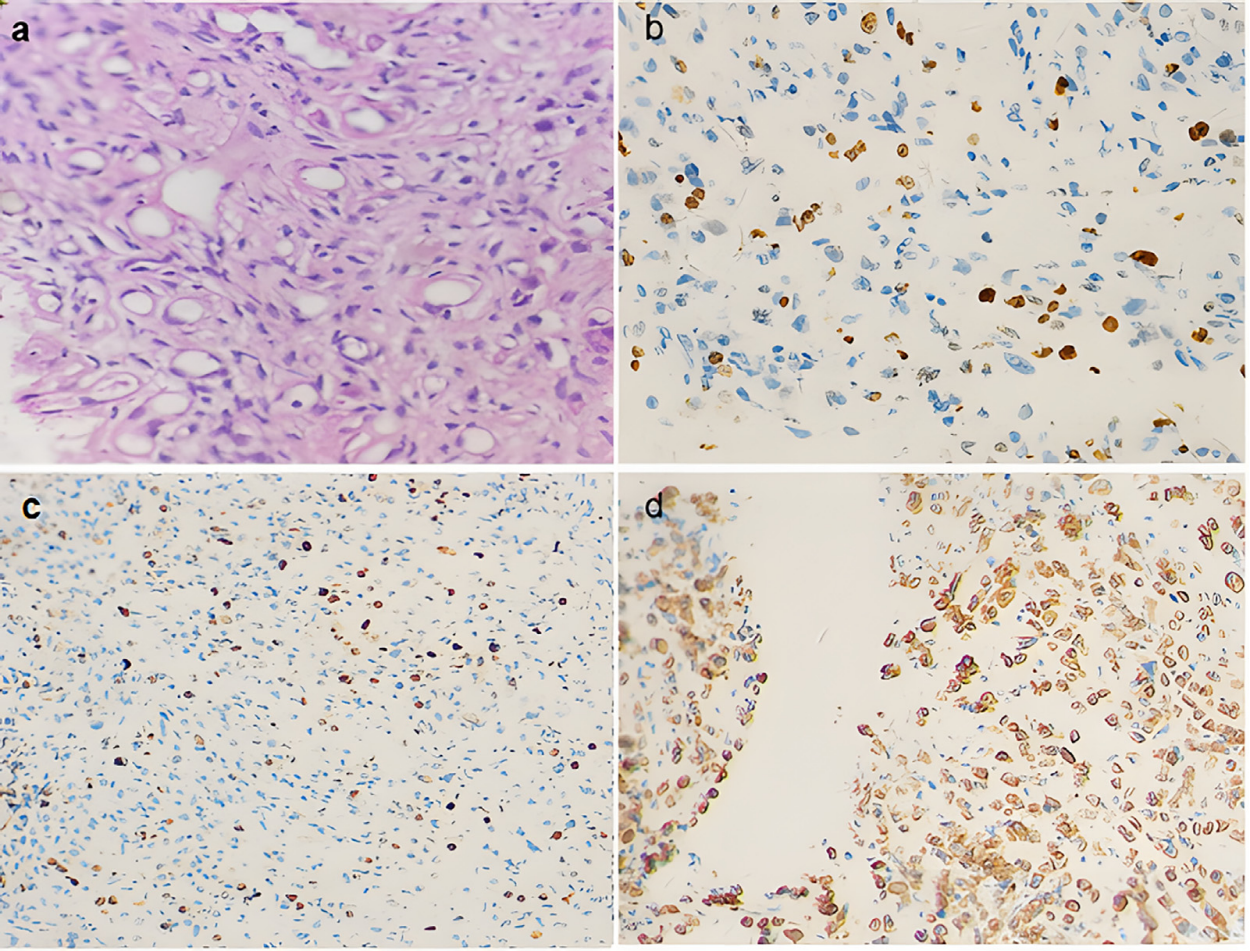
(a) Histology of primary signet-ring cell adenocarcinoma of the bladder. Signet-ring appearance was apparent (HE, ×400); (b‒d) Immunohistochemistry. The signet-ring cell adenocarcinoma was positive for GATA-3 (b), Ki-67 (c) and CEA (d) (×200).

HE staining showed a sparse myxoid background and diffused cancer cells with infiltrative growth. The cancer cells were scattered independently and there were no nest-like structures and no glandular structures. The nuclei were eccentric, and the cytoplasm was pink-stained or partially transparent with a signet-ring-like appearance (Fig. 1a). Immunohistochemical examination showed that the top 3 proteins with positive expression were GATA3 (Fig. 1b), Ki-67 (Fig. 1c) and CEA (Fig. 1d).

### Factors related to survival time in univariate analysis

Univariate COX regression analysis showed that There were statistically significant differences in TNM stage, degree of differentiation, surgical method, whether chemotherapy was administered, whether metastasis was postoperative, and GATA-3 positive expression. The probability of death in patients with SRCC at T3-stage was 8.378 times that at T2-stage, and the probability of death at T4-stage was 23.204 times that at T2-stage; the probability of death at the N1 stage was 3.497 times that at N0-stage, the probability of death at N2-stage was 3.077 times that at N0-stage, and the probability of death at N3-stage was 6.880 times that at N0-stage; the probability of death at M1 was 3.966 times that at M0-stage (p < 0.05 for all). In addition, the probability of death in patients with poorly differentiated SRCC and undifferentiated SRCC was 8.363 and 15.555 times that of patients with moderately differentiated SRCC, respectively. The mortality rate of patients undergoing radical cystectomy and partial cystectomy was 0.207 and 0.308 times that of patients without surgery, respectively. The mortality rate of patients who received chemotherapy was 0.419 times that of patients without chemotherapy, while radiotherapy failed to affect the mortality rate. The mortality rate of patients with metastasis was 4.227 times that of patients without metastasis. Of note, the mortality rate of patients with liver and lung metastasis was 4.417 times that of patients without metastasis, and the mortality rate of patients with metastasis to other sites was 3.720 times that of patients without metastasis. In patients with positive GATA3 expression, the mortality rate was 0.212 times that of patients with negative GATA3 expression (p < 0.05). Other variables (such as age, gender, radiotherapy, tumor site, urine cytological findings, Ki-67, CEA, AB-PAS, RKT20, villin and VEGF) were not associated with the survival of SRCC patients (p > 0.05) (Table 2).

**Table 2:** Univariate COX hazard analysis of factors related to survival.

	p	HR	95% CI for HR	
			Lower	Upper
Age	0.249	1.032	0.978	1.088
Gender	0.272	0.634	0.281	1.431
T2	Ref			
T3	0.044	8.378	1.062	66.078
T4	0.004	23.204	2.809	191.666
N0	Ref			
N1	0.016	3.497	1.267	9.652
N2	0.041	3.077	1.045	9.064
N3	0.022	6.880	1.323	35.783
M	0.003	3.966	1.592	9.880
Mode of surgery	Ref			
Radical cystectomy	0.002	0.207	0.076	0.564
Partial cystectomy	0.034	0.308	0.103	0.917
TURBT	0.980	0.000	0.000	
Chemotherapy	0.039	0.419	0.183	0.957
Radiation	0.134	0.438	0.149	1.288
Subseq metastases	0.005	4.227	1.547	11.552
Sites of metastases	Ref			
Lung or liver	0.005	4.417	1.571	12.421
Other organs	0.042	3.720	1.046	13.227
Urine cytology	0.412	1.454	0.594	3.561
Moderately differentiated	Ref			
Poorly differentiated	0.041	8.363	1.086	64.412
Undifferentiated	0.023	15.555	1.463	165.366
Tumor location	0.153	0.572	0.267	1.230
GATA-3	0.001	0.212	0.083	0.545
Ki-67	0.202	1.652	0.764	3.572
CEA	0.107	0.448	0.169	1.188
AB-PAS	0.096	2.023	0.882	4.639
RKT20	0.938	0.957	0.319	2.876

### Independent factors related to survival time in multivariate analysis

Then, the variables with significant differences in the univariate analysis were included in the multivariate COX regression analysis. Results showed GATA3 and T-stage were two independent prognostic factors of survival. The mortality rate of patients with positive GATA3 expression was 0.328 times that of patients with negative GATA3 expression (95% CI 0.130‒0.830, p = 0.019 < 0.05). The mortality rate of patients with SRCC at T3-stage was 6.487 times that of patients with SRCC at T2-stage (95% CI 0.806‒52.206); the mortality rate of patients with SRCC at T4-stage was 16.374 times that of patients with SRCC at T2 (95% CI 1.883‒142.397) (Table 3).

**Table 3:** Multivariate COX regression analysis of factors related to survival.

		p	HR	95% CI for HR	
				Lower	Upper
Step 1	GATA-3	0.019	0.328	0.130	0.830
	T2	Ref			
	T3	0.039	6.487	0.806	52.206
	T4	0.011	16.374	1.883	142.397

**Fig. 2:**
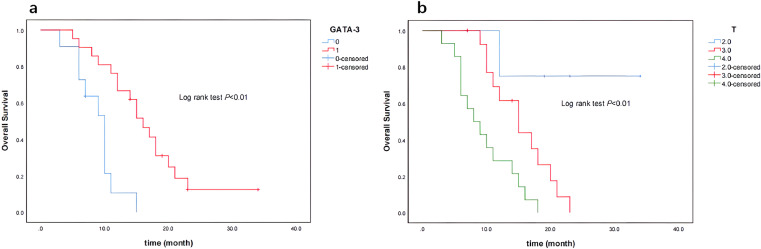
(a) Kaplan-Meier survival curve (GATA-3 expression). Blue, GATA-3 negative group; green lane, GATA-3 positive group. (b) Kaplan-Meier survival curve (T-stage). Blue, T2 group; red, T3 group; green, T4 group.

Of 32 patients, 27 died, and the median survival time was 12 months. In 21 patients positive for GATA-3 expression, 17 patients died with a median survival time of 16 months. In 11 patients negative for GATA3 expression, 10 patients died with a median survival time of 10 months. The Kaplan-Meier survival curve showed GATA3-positive patients had a better prognosis (p < 0.01, Fig. 2a). In 3 patients with SRCC at T2-stage, none died; in 15 patients with SRCC at T3-stage, 13 patients died with a median survival time of 13 months. All the 14 patients with SRCC at T4-stage died with a median survival time of 8 months. As shown by the Kaplan-Meier survival curve, patients with SRCC at T2-stage had the best prognosis, followed by patients with SRCC at T3-stage, and patients with T4-stage SRCC had the worst prognosis (p < 0.01) (Fig. 2b).

## Discussion

Primary SRCC of the bladder is an extremely rare malignancy and was first reported by Saphir in 1955.^2^ In 1996, Holmäng et al.^11^ investigated 10 patients with primary SRCC of the bladder based on the analysis of a database involving 1529 bladder cancer patients in Sweden. In 1991, Grignon et al.^12^ investigated 11 patients with primary SRCC of the bladder in the MD Anderson Cancer Center's database. To date, few studies with large sample sizes have been conducted to investigate the SRCC of the bladder due to its low incidence. The clinical manifestations of primary SRCC of the bladder are not significantly different from those of other bladder malignant tumors. Ozeki et al.^13^ reported that dysuria, frequent micturition, urinary incontinence, and urinary retention were also the symptoms of primary SRCC of the bladder. Shusuke et al.^3^ investigated 57 patients with primary SRCC of the bladder, and reported painless macroscopic hematuria in 63% of patients and bladder irritation symptoms in 46%, and dysuria and urinary incontinence were also common clinical manifestations. In the present study, 19 (59%) of the 32 patients had macroscopic hematuria, and 8 (25%) developed bladder irritation symptoms, which were consistent with previously reported.

Currently, there is still no consensus on the early diagnosis of primary SRCC of the bladder. Grignon et al.^12^ reported that 47.1% of patients had no discernible neoplasms on cystoscopy. Wang et al.^4^ reported that primary SRCC of the bladder was frequently found at the top and anterior walls of the bladder. In the present study, SRCC at the top and anterior walls of the bladder accounted for only 25%, which also suggests that the location of SRCC in the bladder is non-specific. As reported, SRCC of the bladder displays diffused and infiltrative growth, and thus the neoplastic lesions are often unrecognizable at an early stage of SRCC on cystoscopy. When clinical symptoms are present and abnormalities are observed on cystoscopy, SRCC of the bladder is often diagnosed at T3-stage or more malignant, which may result in a poor response to treatments and a poor prognosis.

Of note, the primary SRCC of the bladder should be differentiated from the metastatic SRCC of the bladder. In patients with metastatic SRCC of the bladder, the primary site of SRCC can be identified on clinical examinations, and the gastrointestinal tract is a common site of primary SRCC. Once a patient develops bloody stool, abdominal pain, nausea, vomiting, and other gastrointestinal symptoms, the metastatic SRCC of the bladder should be suspected, and the contrast-enhanced CT of the abdomen and pelvis and gastrointestinal endoscopy may be employed for the clinical diagnosis and differential diagnosis. In addition, immunohistochemistry is an important tool to identify the source and type of cancer cells. Currently, there is no widely accepted consensus on the immunohistochemical features of primary SRCC of the bladder. Kunze et al.^14^ reported that primary SRCC of the bladder was positive for pS2 and MUC5AC; Del Sordo et al.^15^ reported the positive expression of RKT7 (3/5), RKT20 (2/5), β-catenin (5/5), CK34BE12 (2/5), CEA (4/5), and villin (1/5) in a study on 5 patients with SRCC of the bladder, but the cancer tissues were negative for HepPar1, p63, PSA, PSAP, thrombomodulin and TTF-1. Thomas et al.^16^ found positive expression of RKT20, RKT7, CDX2, β-catenin, E-cadherin and villin in 78%, 67%, 33%, 78%, 67% and 100% of patients with SRCC of the bladder, respectively. In the present study, positive expression was mainly found in the GATA3 (72%), Ki-67 (63%), CEA (44%), AB-PAS (34%) and RKT20 (28%), and the cancer tissues were negative for E-cadherin, CDX2, ER and PR.

To date, surgery is the preferred treatment for primary SRCC of the bladder. In this study, 43.75% of patients received radical cystectomy, 18.75% underwent partial cystectomy, and 6.25% received TURBT. In addition, 34.4% of patients underwent conservative treatment, because there was a risk for surgery when the SRCC of the bladder was diagnosed at the advanced stage and patients usually have several pre-existing diseases. Primary SRCC of the bladder has a low incidence and thus few randomized, controlled studies have been conducted to investigate the clinical manifestations, diagnosis, and treatments, and therefore there is no consensus on these issues. Fujita et al.^17^ reported that radical cystectomy plus ileal bladder replacement was the treatment of choice for SRCC. The post-operative treatments for SRCC of the bladder are currently in the exploratory stage, and results are still conflicting in some studies. Akamatsu et al.^3^ proposed that intravesical irrigation with Bacille Calmette-Guérin (BCG) was not beneficial for SRCC; several studies have reported that chemotherapy is not recommended for SRCC because of “chemotherapy resistance”.^16^^,^^18^ Reddy et al.^19^ recommended adjuvant radiotherapy and chemotherapy for patients with SRCC of the bladder after surgery, because they could achieve local disease control and reduce the risk of recurrence. Hamakawa et al.^10^ reported a patient who was diagnosed with SRCC of the bladder at T3bN0M0 and received combined chemotherapy with cisplatin + S-1 for three courses after surgery, and recurrence and metastasis were not found within 90 months after surgery. Cobo-Dols et al.^20^ reported a patient with SRCC at pT4aN0M0 was treated with cisplatin and gemcitabine, and recurrence was not observed within 8 months after surgery. Ota et al.^21^ reported that intra-arterial injection of methotrexate and cisplatin combined with radiotherapy was effective for SRCC of the bladder after surgery. Unfortunately, there is no standardized regimen for the chemotherapy of SRCC of the bladder.

At the early stage, primary SRCC of the bladder has no specific manifestations and even progresses rapidly. In about 46% of patients, primary SRCC of the bladder is initially diagnosed at stage IV. Although it has been reported that the longest tumor-bearing survival time is 16 years, most patients will die within the first year after diagnosis.^3^ In the present study, the 1-year and 2-year survival rates of patients with SRCC of the bladder were 53.1%, and 9.4%, respectively, which was consistent with previously reported. The COX proportional hazards model was employed to analyze variables related to the survival of patients with primary SRCC of the bladder in the present study. Univariate analysis showed that TNM stage, cancer differentiation, and metastasis after treatment were risk factors for prognosis in SRCC patients, while surgery, chemotherapy, and positive GATA3 expression were protective for the prognosis of SRCC patients. Multivariate analysis showed that GATA3 expression was an independent protective factor for the prognosis, but T-stage was an independent risk factor for the survival of patients with primary SRCC of the bladder. There is evidence showing that increased CEA after surgery in SRCC patients is closely related to the disease progression, and thus CEA is recommended to determine the degree of malignancy and monitor the progression of SRCC.^22^ However, in the present study, CEA remained stable in the follow-up period.

## Conclusions

In conclusion, primary SRCC of the bladder is a rare malignancy and has no specific clinical manifestations and imaging findings at an early stage. Moreover, it is highly malignant with rapid progression, shows infiltrative growth, and is more likely to metastasize at an early stage, resulting in a poor prognosis. This study indicates T-stage is an independent risk factor for survival, and positive GATA3 expression serves as an independent protective factor for prognosis in patients with primary SRCC of the bladder. Radical cystectomy is currently accepted as the treatment of choice for primary SRCC of the bladder, and postoperative radiotherapy, chemotherapy or immunotherapy may be promising to improve the prognosis of these patients.

## Authors’ contributions

Conceptualization: Xin Hong and Tian Wang; Data curation: Xin Hong, Tian Wang, Qing Liu, Jianlong Bi, and Hui Li; Formal analysis: Xin Hong and Tian Wang; Investigation: Xin Hong, Tian Wang, Qing Liu, Jianlong Bi, and Hui Li; Methodology: Xin Hong, Tian Wang, Qing Liu, Jianlong Bi, and Hui Li; Project administration: Xin Hong; Resources: Xin Hong, Tian Wang, Qing Liu, Jianlong Bi, and HL; Software: Xin Hong; Supervision: Xin Hong; Validation: Xin Hong; Visualization: Xin Hong; Roles/Writing-original draft: Xin Hong and Tian Wang; Writing-review & editing: Xin Hong.

## Declaration of competing interest

The authors declare no conflicts of interest.
